# Supplementary figures and images for: Comparison of soil quality assessment methods for different vegetation eco-restoration techniques at engineering disturbed areas
Source: PeerJ. 2024 Sep 5;12:e18033. doi: 10.7717/peerj.18033 (PMC11380839; doi:10.7717/peerj.18033)

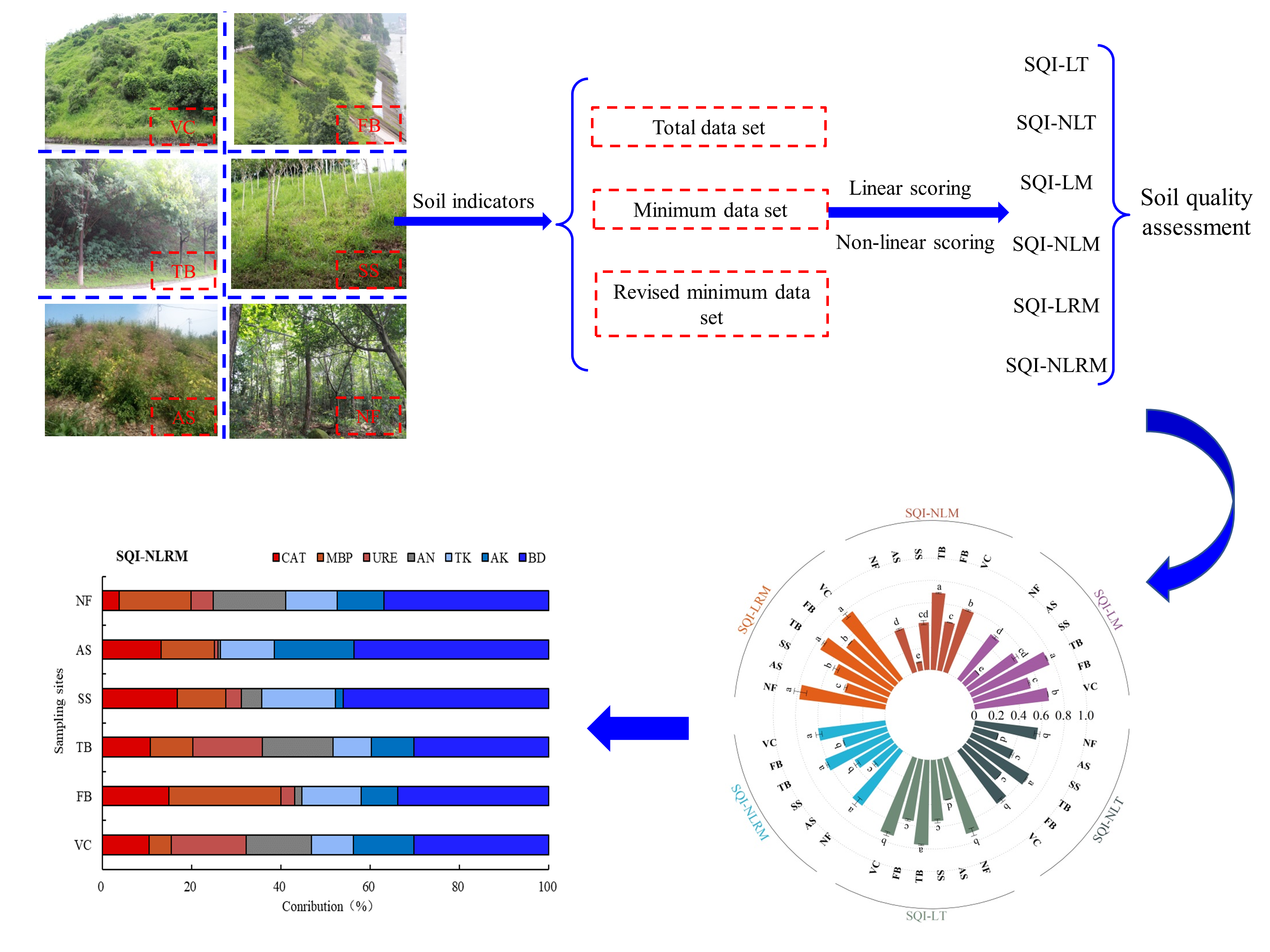

Supplement: Supplemental Information 2 — Photo credit: Xia Dong [file peerj-12-18033-s002.png]

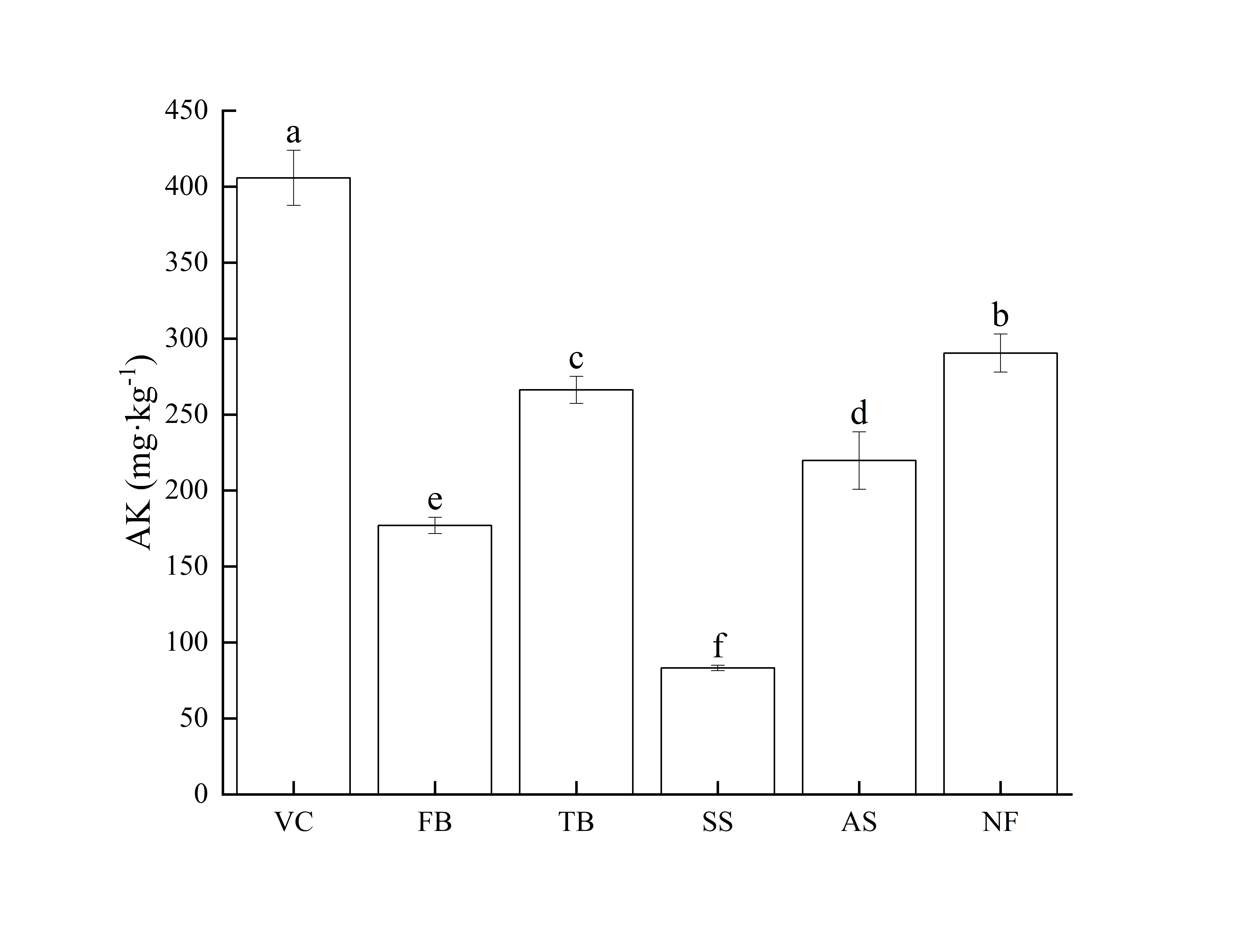

Supplement: Supplemental Information 3 [file peerj-12-18033-s003.jpg]

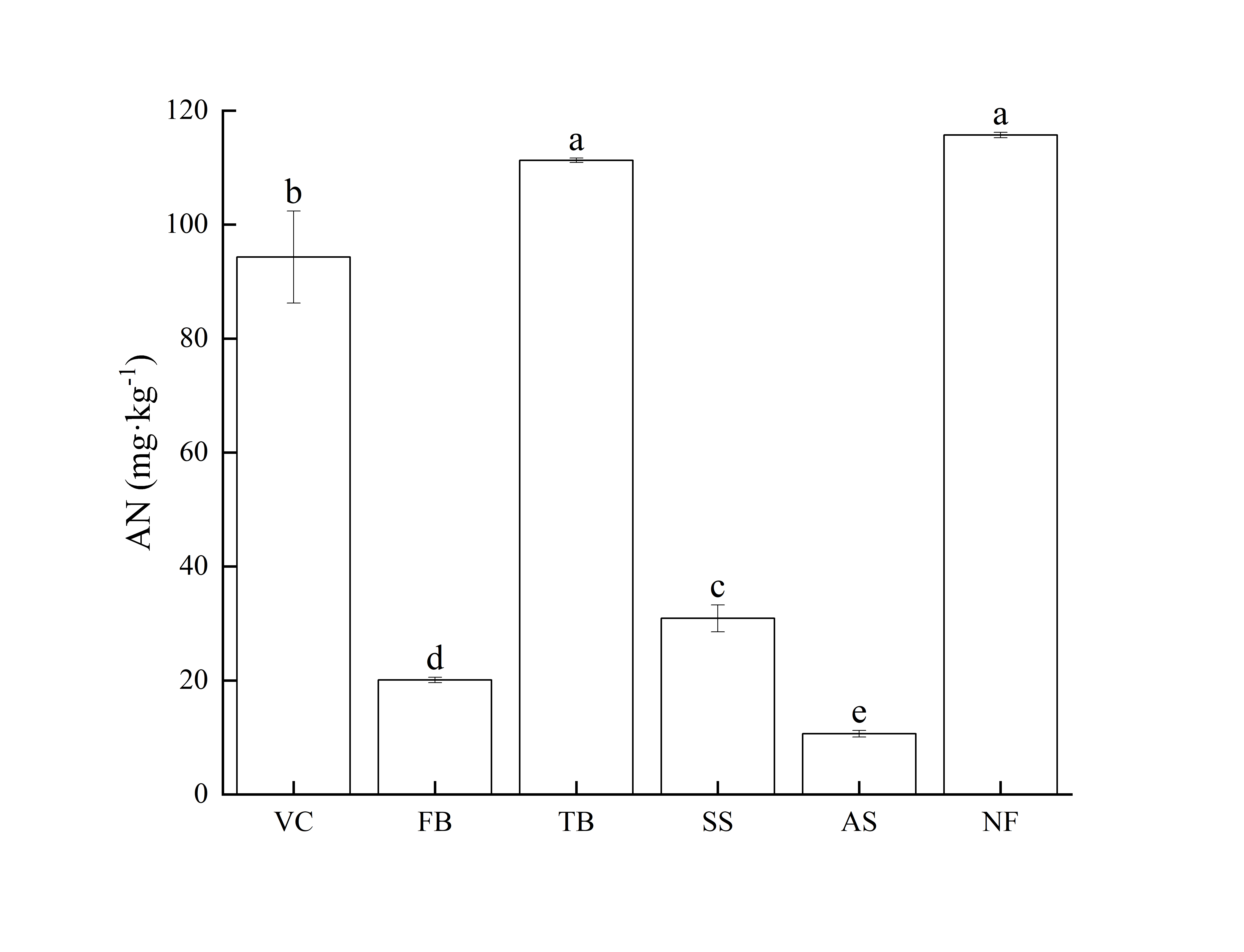

Supplement: Supplemental Information 4 [file peerj-12-18033-s004.jpg]

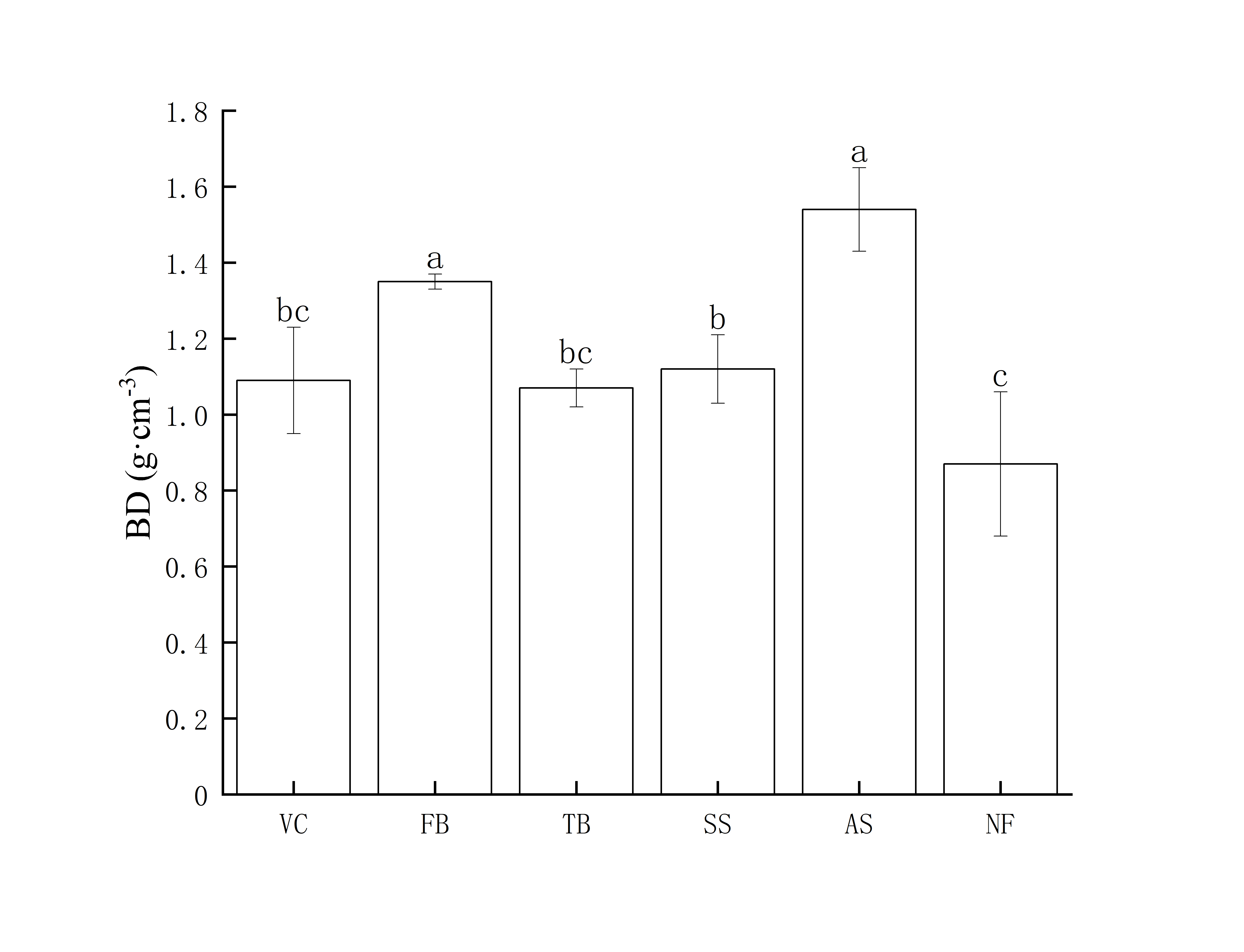

Supplement: Supplemental Information 5 [file peerj-12-18033-s005.jpg]

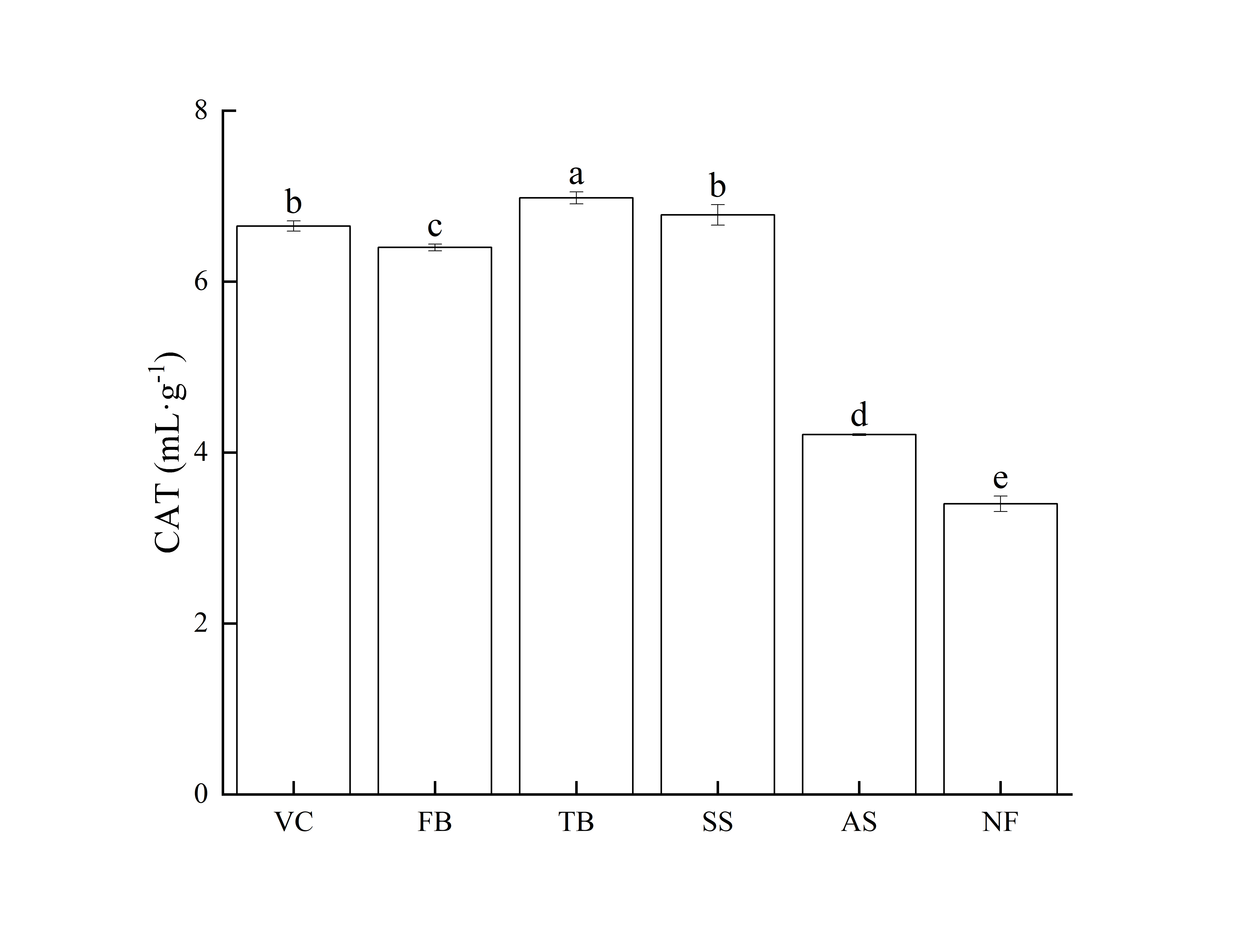

Supplement: Supplemental Information 6 [file peerj-12-18033-s006.jpg]

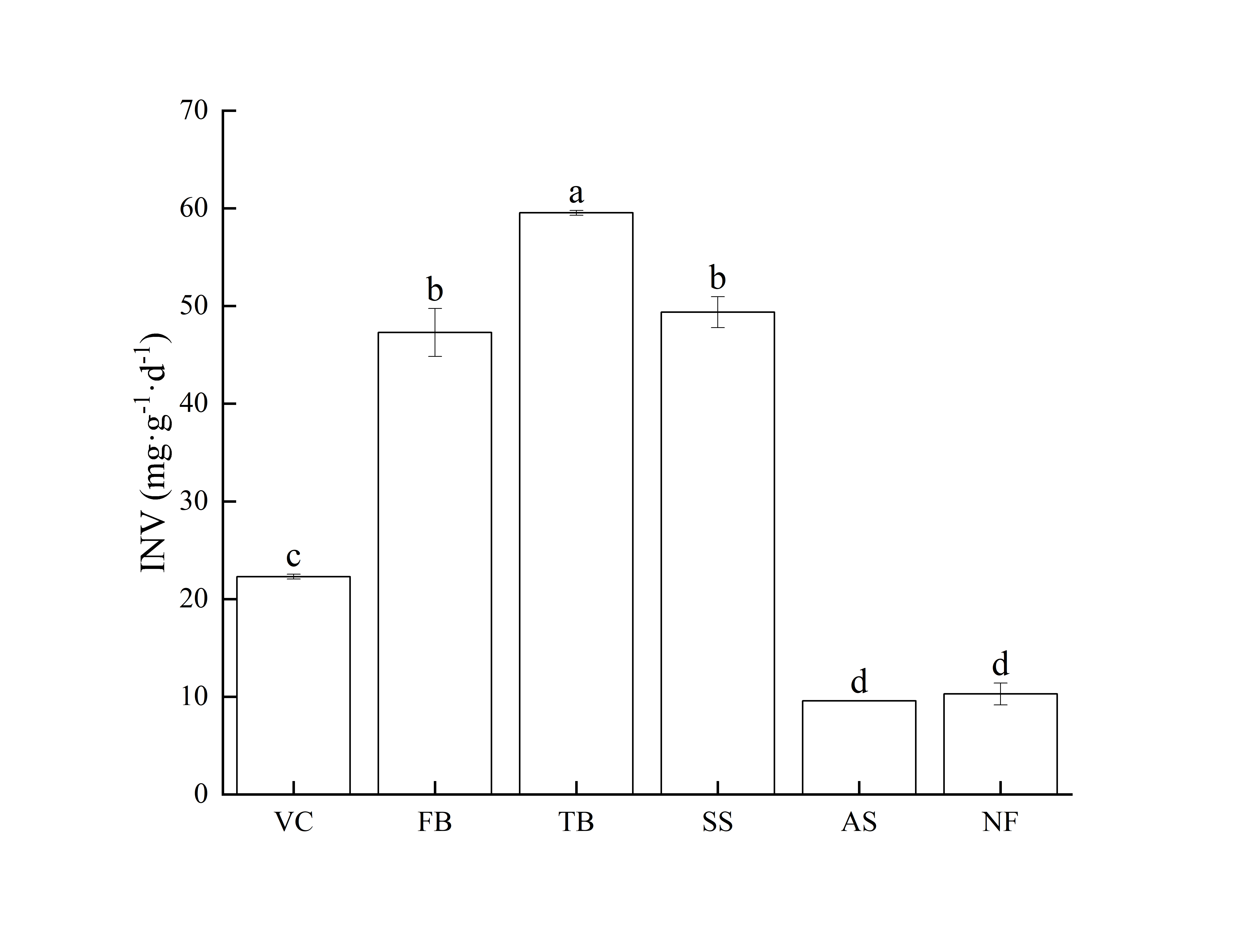

Supplement: Supplemental Information 7 [file peerj-12-18033-s007.jpg]

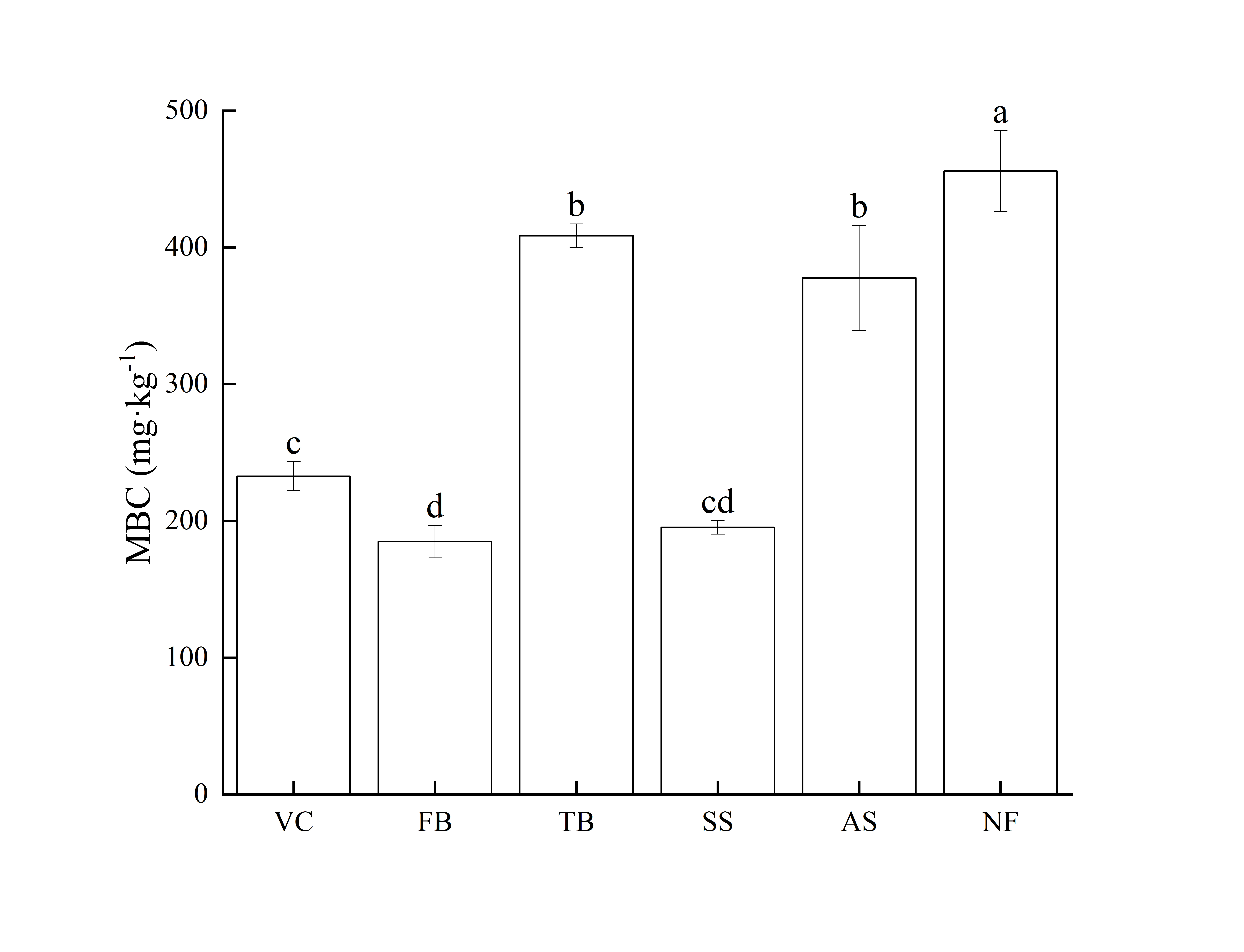

Supplement: Supplemental Information 8 [file peerj-12-18033-s008.jpg]

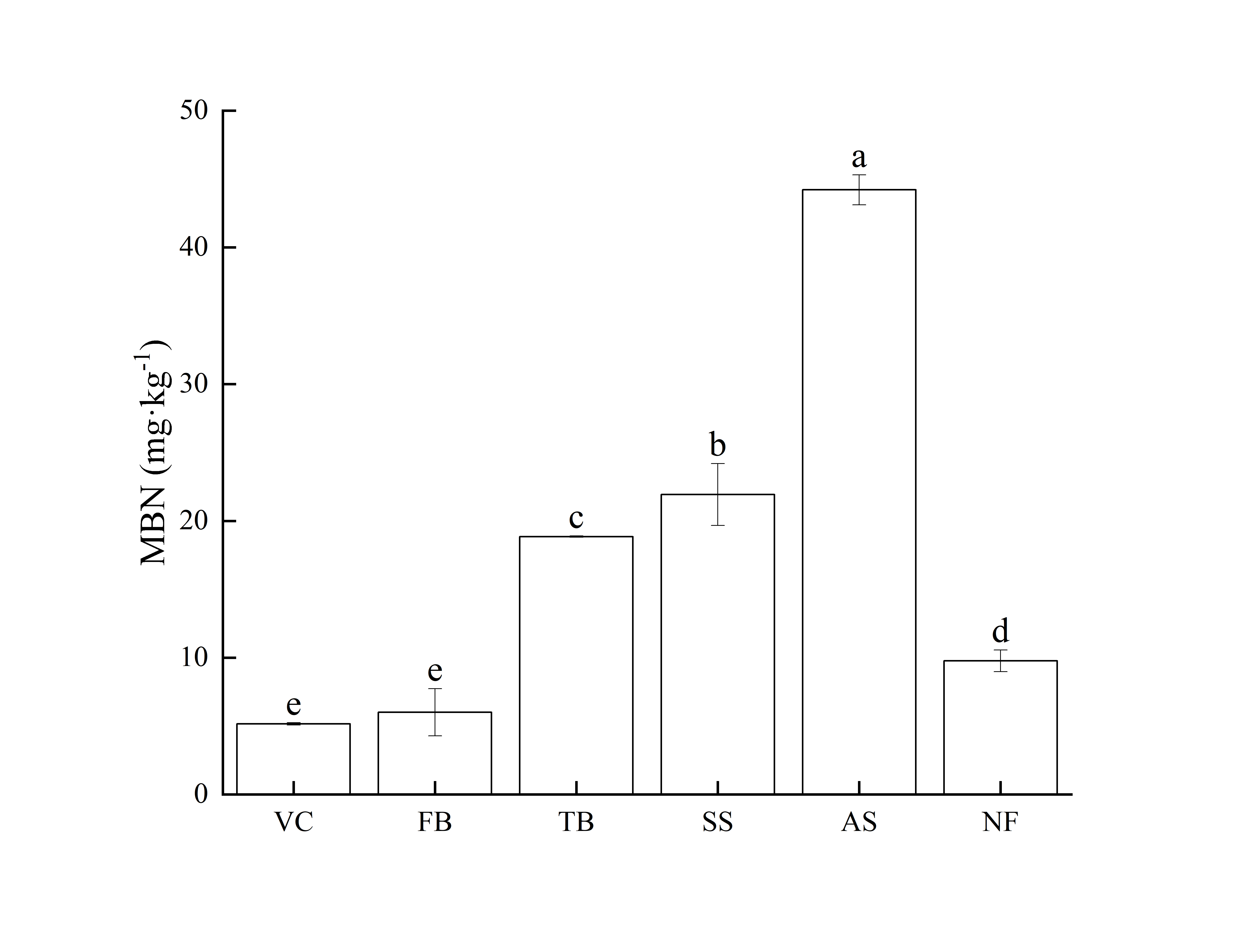

Supplement: Supplemental Information 9 [file peerj-12-18033-s009.jpg]

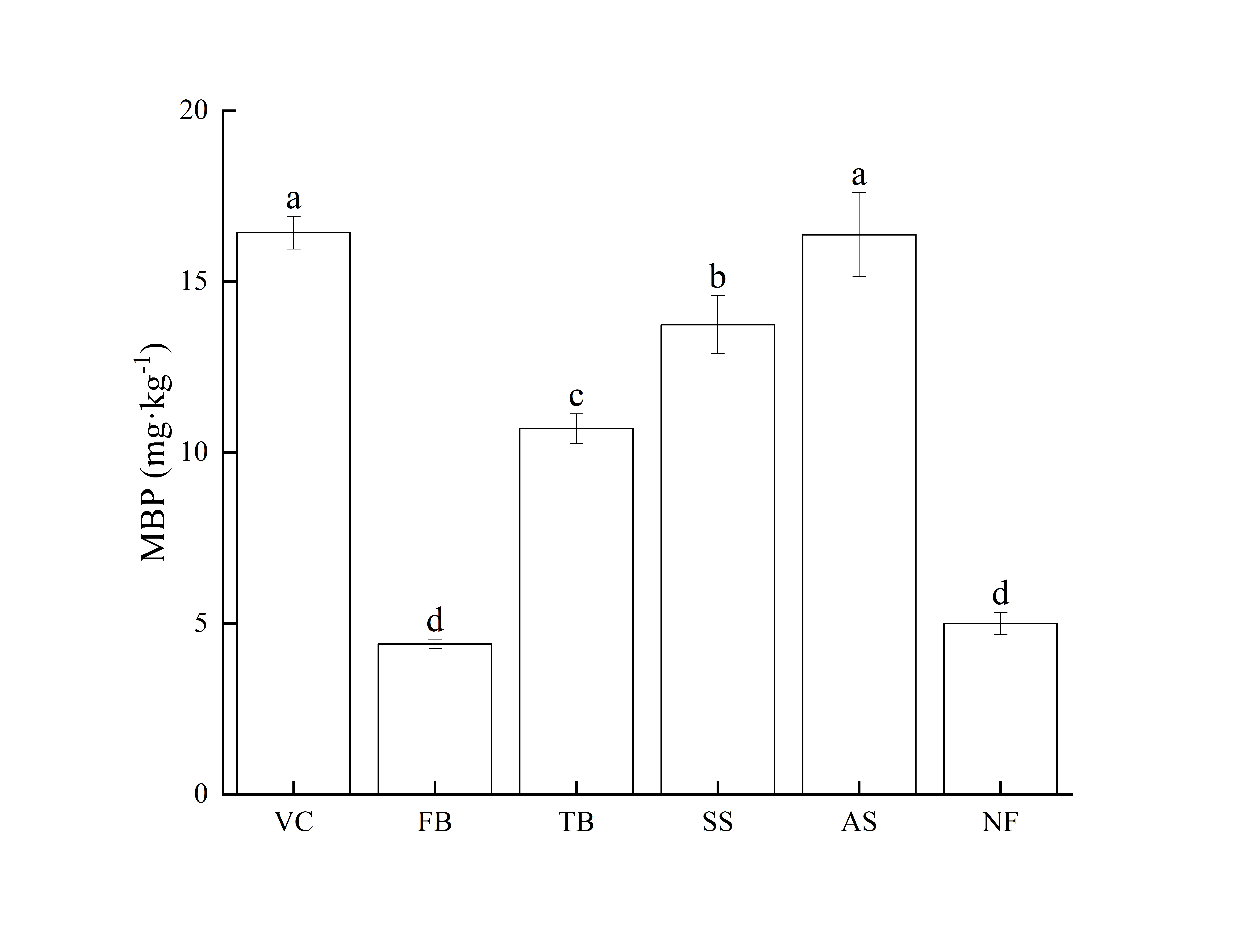

Supplement: Supplemental Information 10 [file peerj-12-18033-s010.jpg]

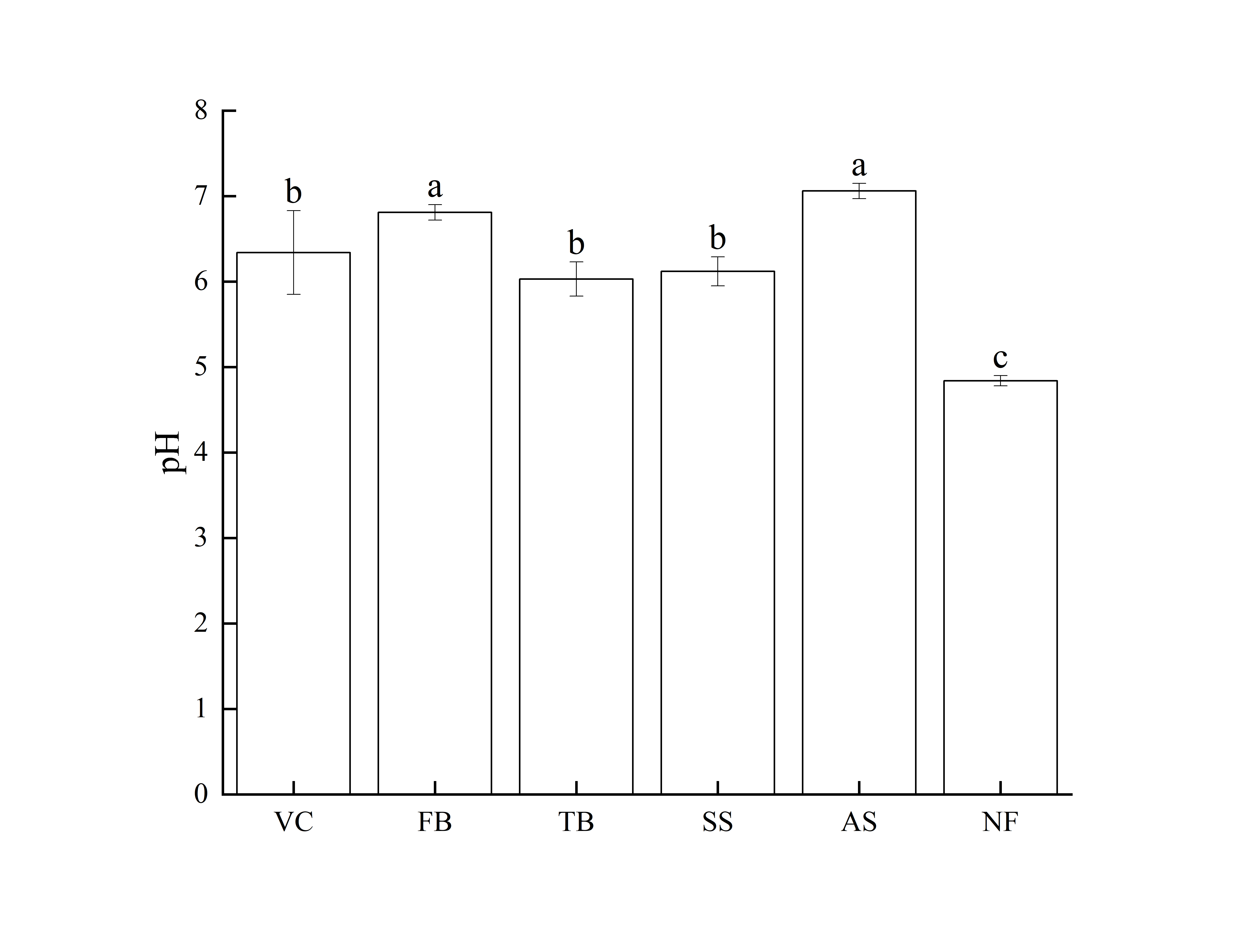

Supplement: Supplemental Information 11 [file peerj-12-18033-s011.jpg]

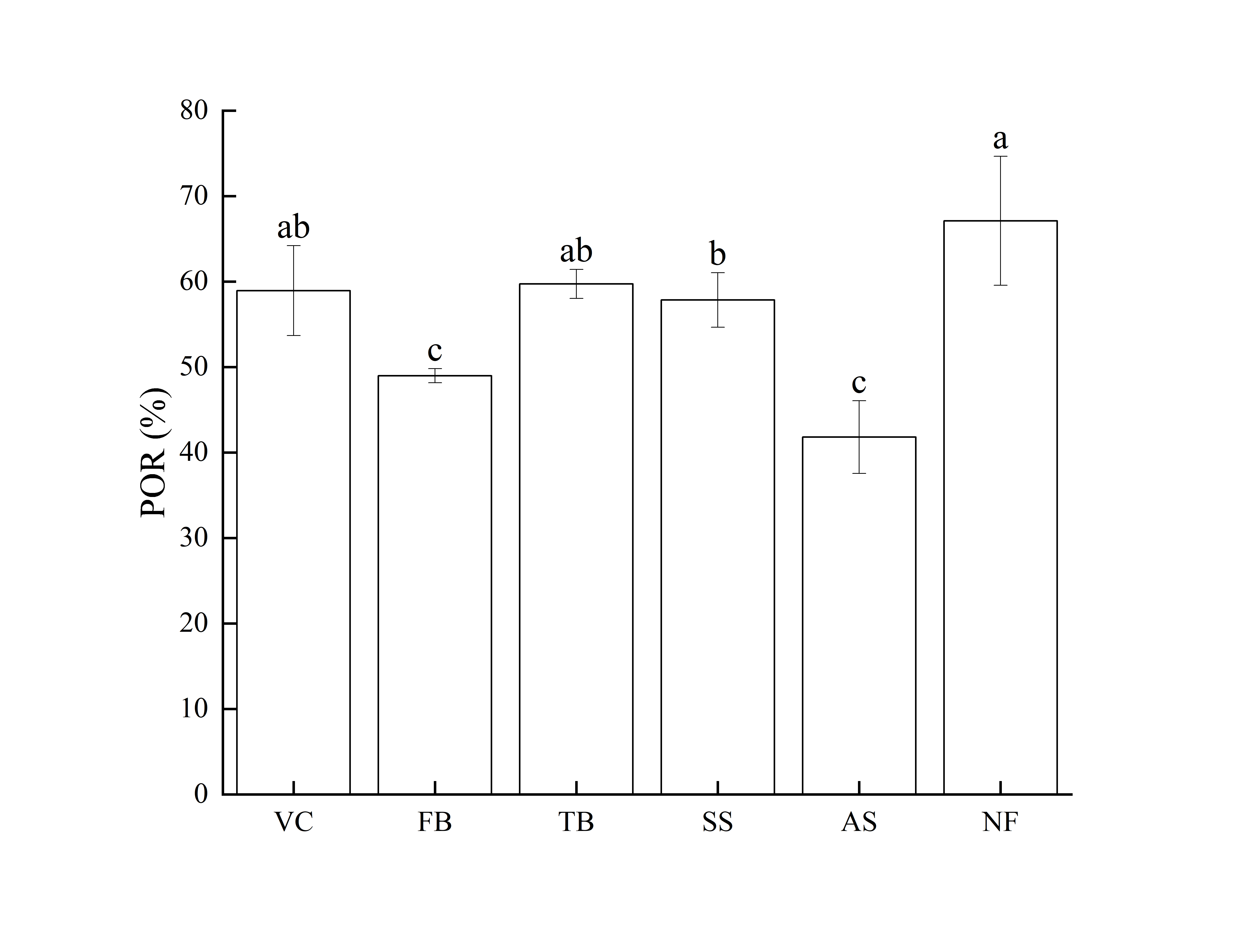

Supplement: Supplemental Information 12 [file peerj-12-18033-s012.jpg]

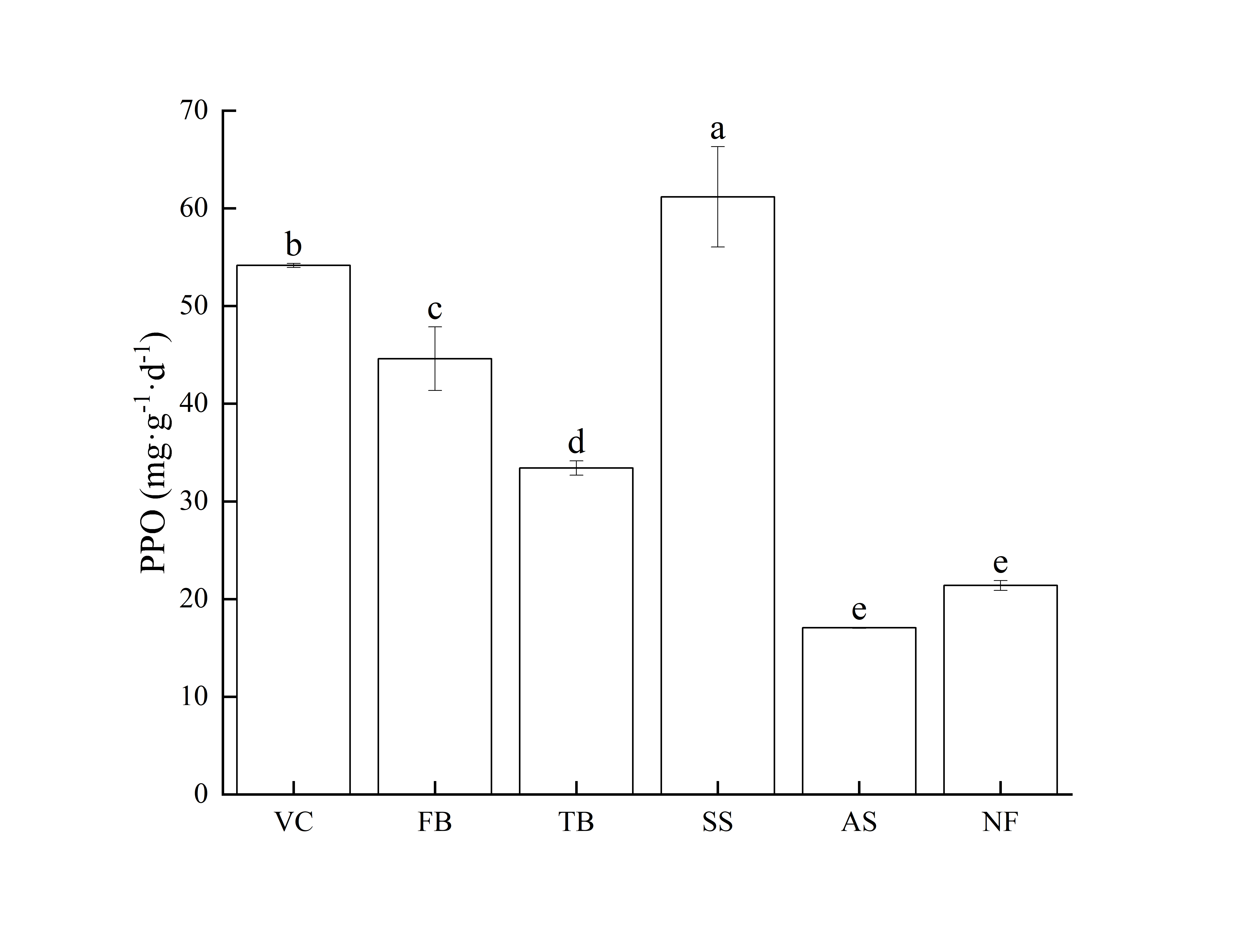

Supplement: Supplemental Information 13 [file peerj-12-18033-s013.jpg]

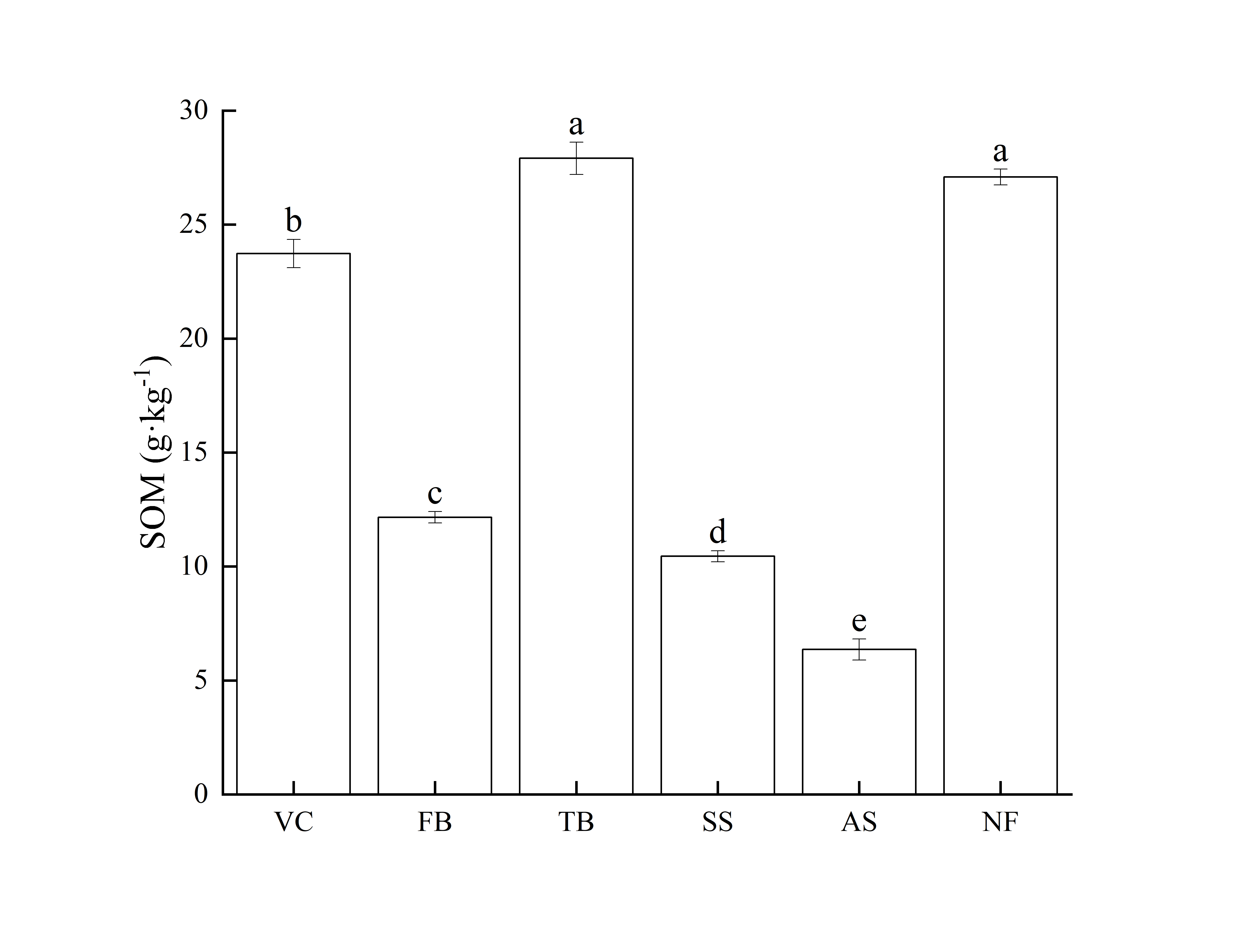

Supplement: Supplemental Information 14 [file peerj-12-18033-s014.jpg]

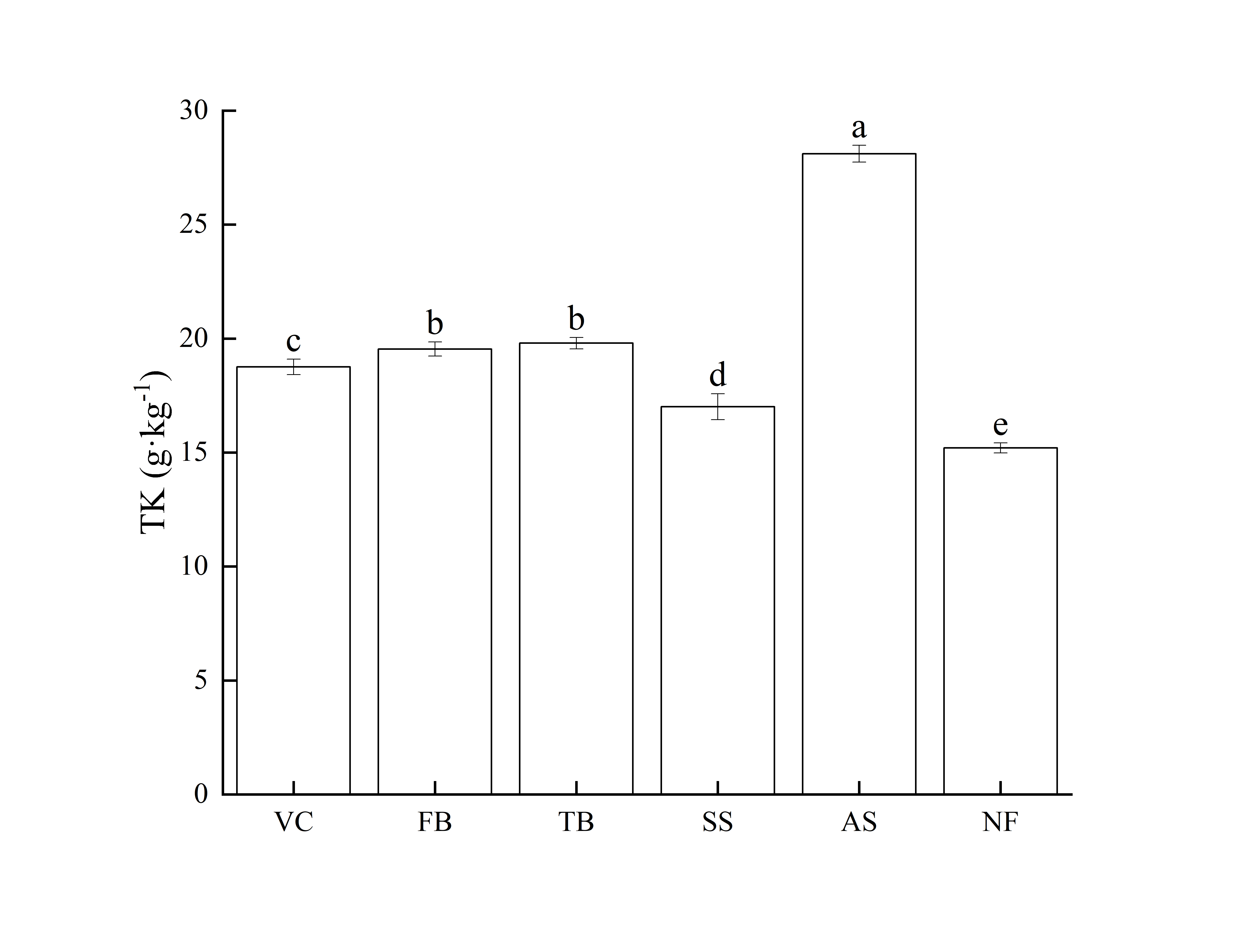

Supplement: Supplemental Information 15 [file peerj-12-18033-s015.jpg]

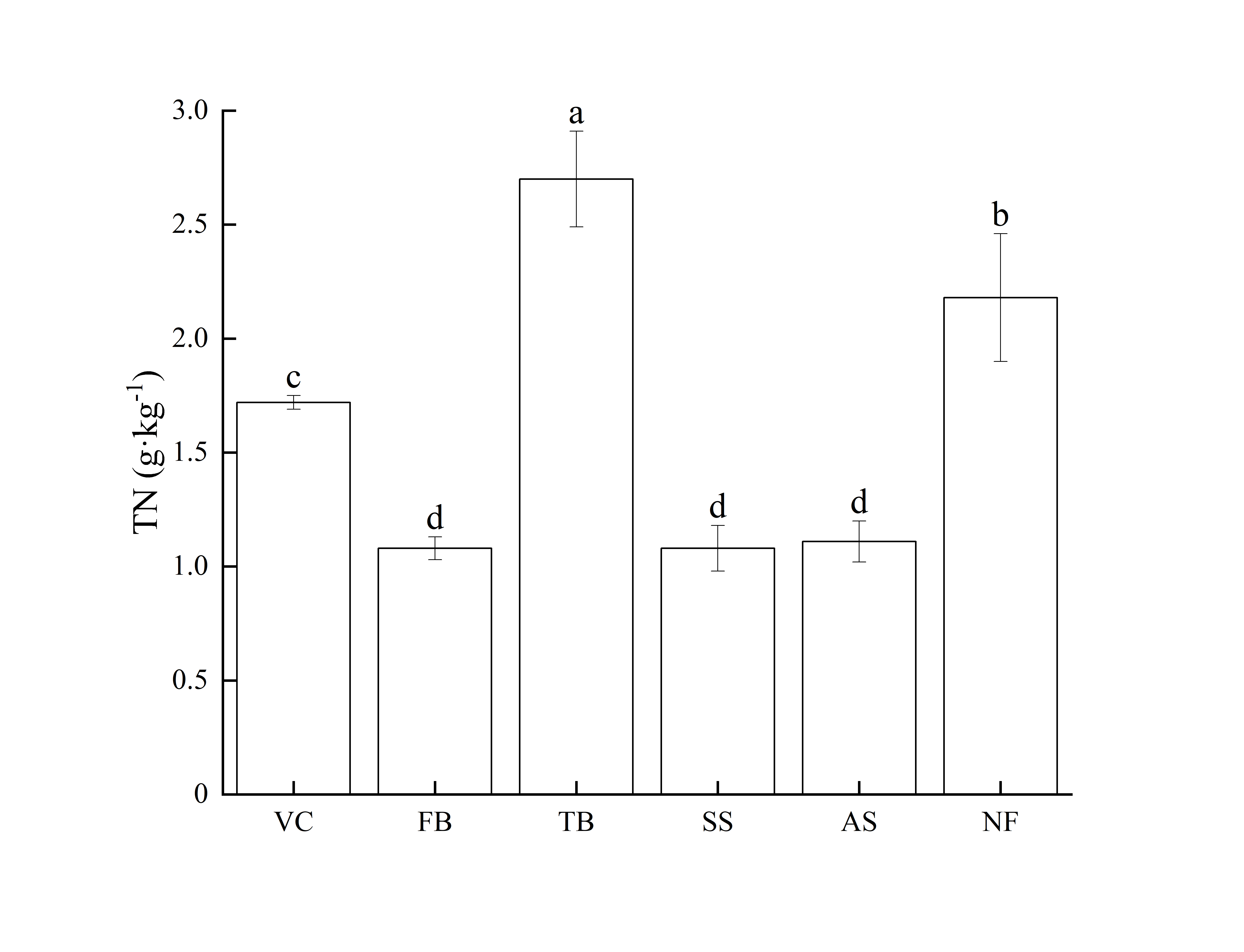

Supplement: Supplemental Information 16 [file peerj-12-18033-s016.jpg]

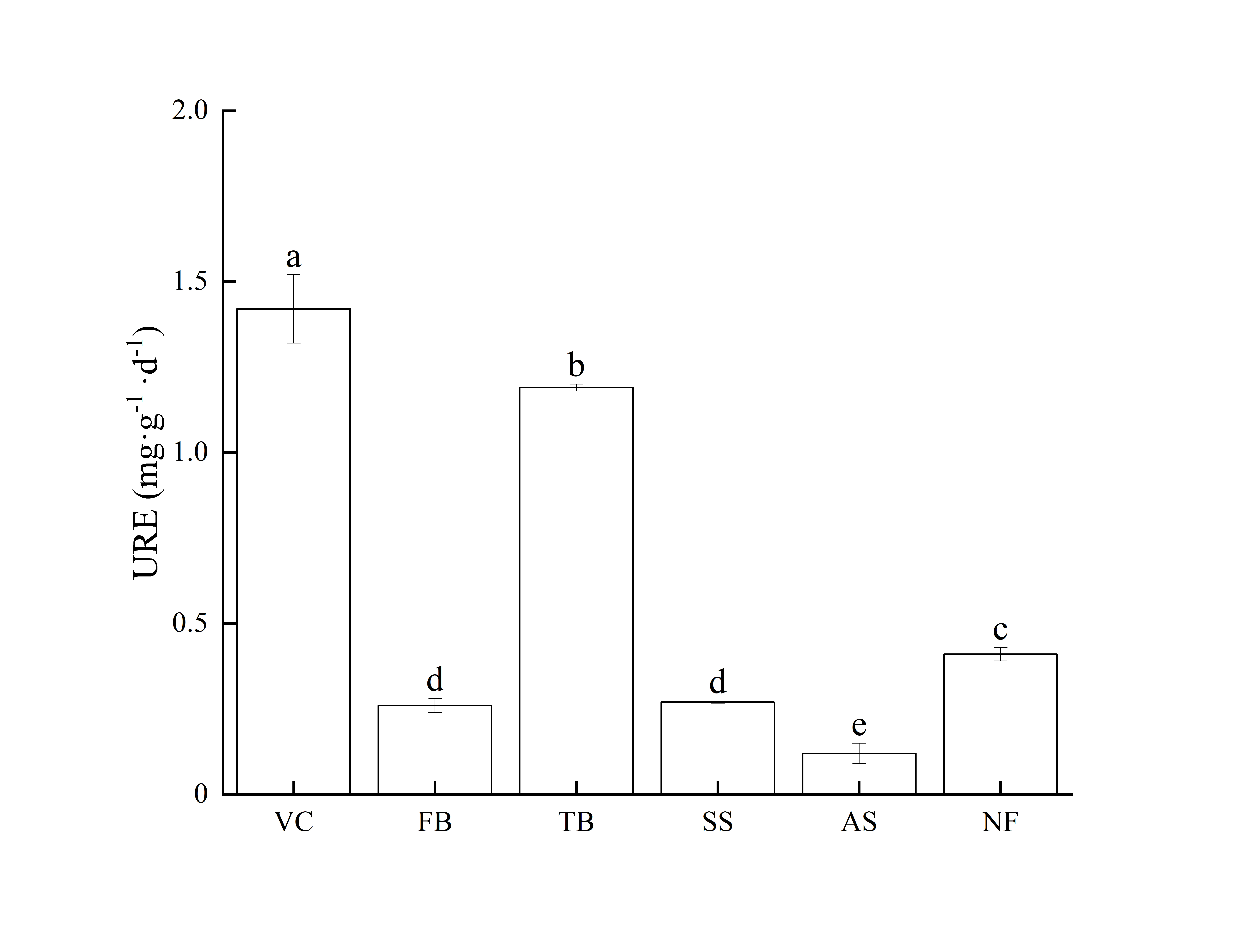

Supplement: Supplemental Information 17 [file peerj-12-18033-s017.jpg]

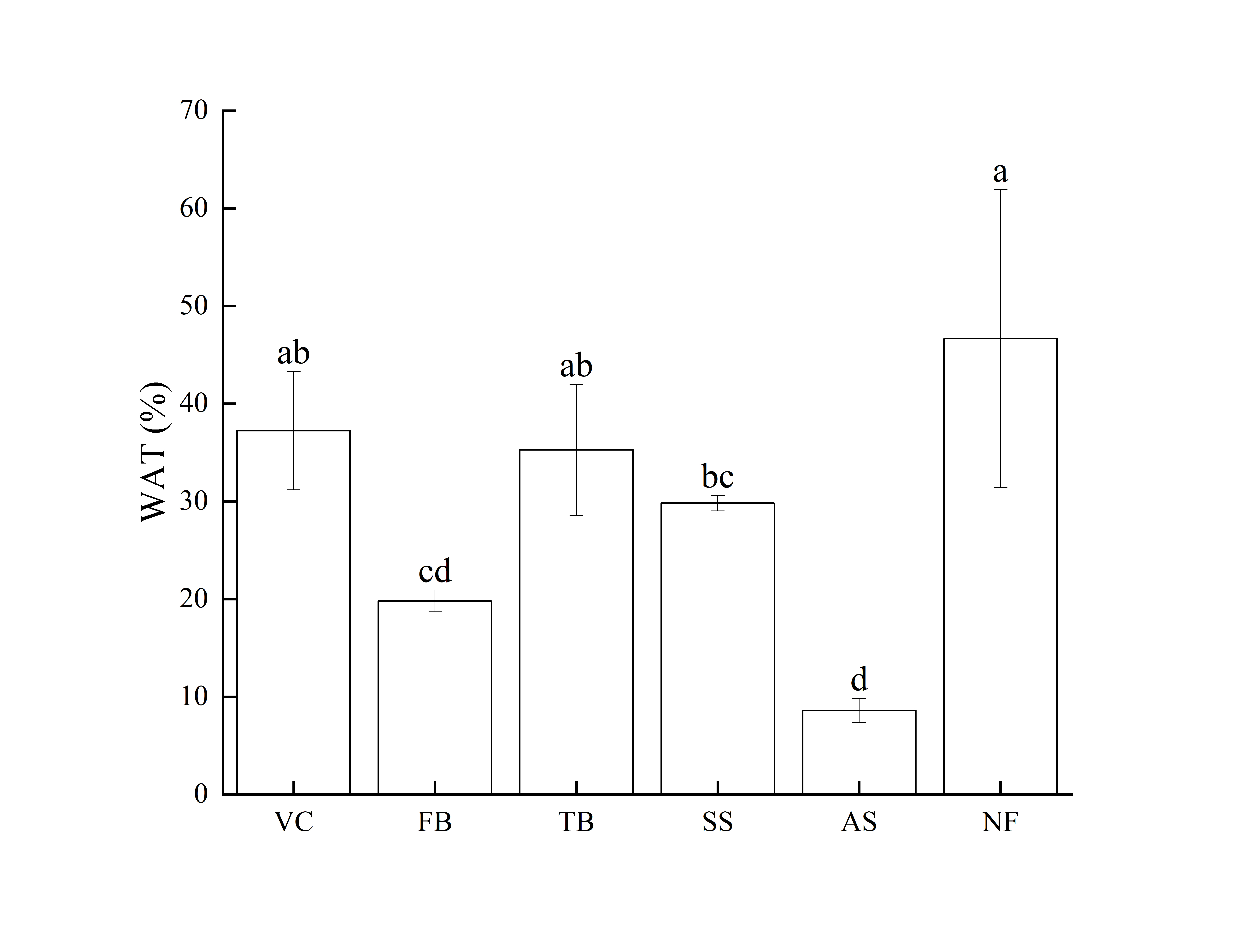

Supplement: Supplemental Information 18 [file peerj-12-18033-s018.jpg]

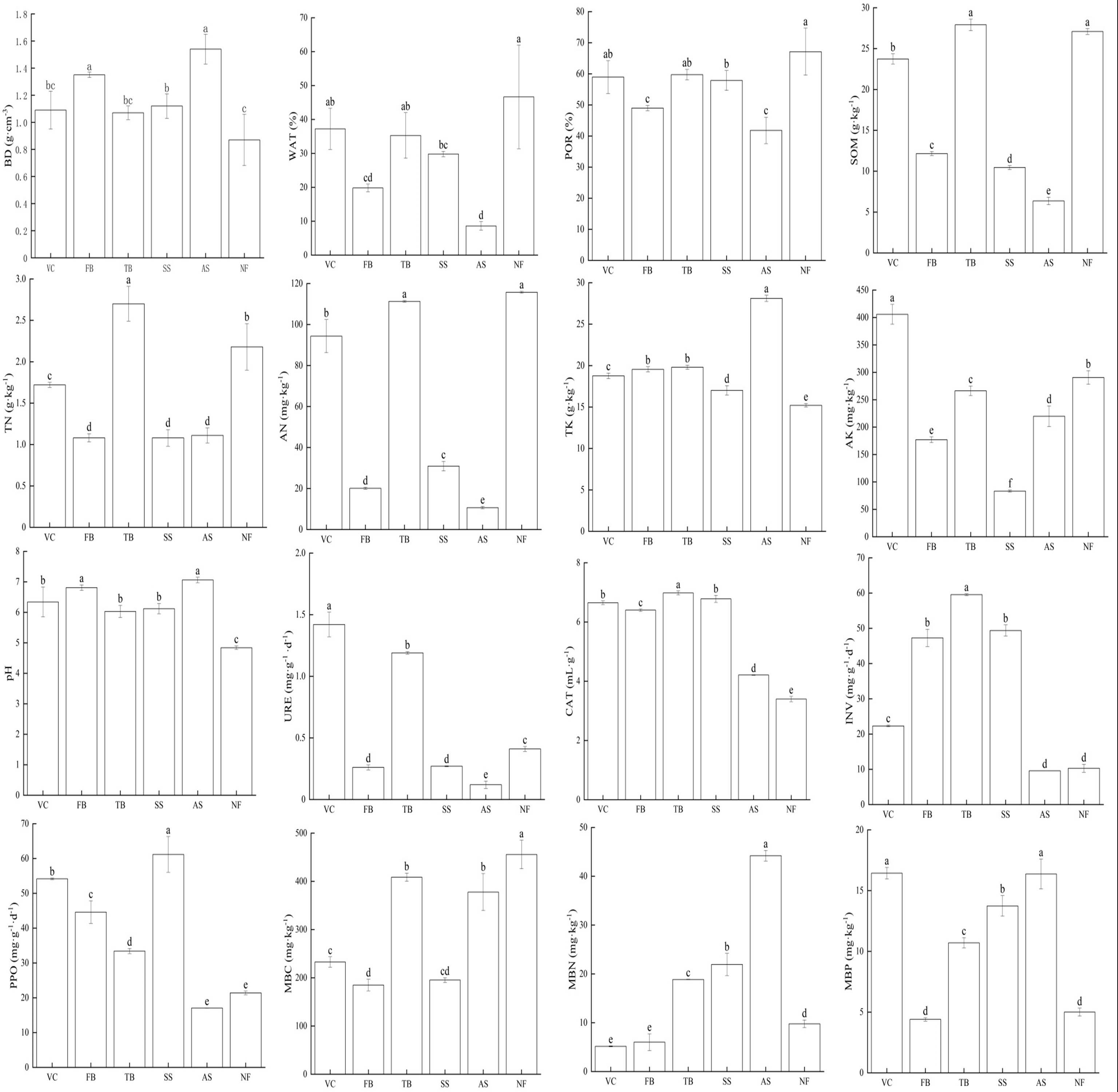

Supplement: Supplemental Information 19 [file peerj-12-18033-s019.jpg]
